# Entropy Modifications of Charged Accelerating Anti-de Sitter Black Hole

**DOI:** 10.3390/e27090900

**Published:** 2025-08-25

**Authors:** Cong Wang, Jie Zhang, Shu-Zheng Yang

**Affiliations:** 1 College of Physics and Electronic Engineering, Qilu Normal University, Jinan 250300, China; 2 School of Arts and Sciences, Shanghai Dianji University, Shanghai 200240, China; zhangjie@126.com; 3 College of Physics and Astronomy, China West Normal University, Nanchong 637002, China; szyangcwnu@126.com

**Keywords:** quintessence, Anti-de Sitter black hole, Lorentz-breaking, quantum tunneling radiation, Bekenstein–Hawking entropy

## Abstract

The Lorentz-breaking theory not only modifies the geometric structure of curved spacetime but also significantly alters the quantum dynamics of bosonic and fermionic fields in black hole spacetime, leading to observable physical effects on Hawking temperature and Bekenstein–Hawking entropy. This study establishes the first systematic theoretical framework for entropy modifications of charged accelerating Anti-de Sitter black holes, incorporating gauge-invariant corrections derived from Lorentz-violating quantum field equations in curved spacetime. The obtained analytical expression coherently integrates semi-classical approximations with higher-order quantum perturbative contributions. Furthermore, the methodologies employed and the resultant conclusions are subjected to rigorous analysis, establishing their physical significance for advancing fundamental investigations into black hole entropy.

## 1. Introduction

This study aims to investigate the thermodynamic properties of charged accelerating Anti-de Sitter (AdS) black holes and to elucidate the physical implications of entropy corrections within the Lorentz-breaking theoretical framework. To deepen our understanding of black hole entropy and its physical implications, it is essential to first clarify the methodology and definition of black hole entropy research. When treating a black hole as a thermodynamic system, its entropy is closely related to the Hawking temperature at the event horizon, as dictated by the first law of black hole thermodynamics. From a quantum theoretical perspective, the entropy is found to be proportional to the area of the event horizon, measured in Planck units ($\propto l_{p}^{2}$). This suggests that black hole thermodynamics encompasses quantum gravitational effects that warrant further exploration. Additionally, the black hole area theorem states that the surface area of a black hole never decreases in the forward time direction, which is a direct manifestation of the second law of black hole thermodynamics and serves as an important physical demonstration of the “arrow of time” in gravitational physics. Since ‘t Hooft proposed the brick wall model in 1985, research into black hole entropy has significantly advanced [1]. This model posits that the entropy of a black hole is equivalent to the entropy of a quantum gas in thermal equilibrium outside the black hole. Subsequently, Zhao and colleagues improved ‘t Hooft’s brick wall model by developing the membrane paradigm [2,3,4,5], which enables the calculation of entropy for non-stationary black holes and static or stationary black holes in non-thermal equilibrium states. The membrane paradigm reveals that black hole entropy fundamentally originates from the entropy of a two-dimensional membrane located at the black hole’s surface (event horizon). Specifically, it represents the entropy of a two-dimensional surface formed by the intersection of a null hypersurface with spacelike hypersurfaces in three-dimensional space. This entropy arises from contributions of a quantum gas on the two-dimensional membrane and can be interpreted as the entropy of quantum states on the two-dimensional horizon. The membrane paradigm is applicable to studying the entropy of static, stationary, and non-stationary black holes. Research has demonstrated that black hole entropy is proportional to the area of the event horizon. General relativity and quantum field theory are both founded on Lorentz symmetry [6,7,8]. However, these theories cannot be unified at high energies due to the inherent non-renormalizability of Einstein’s general relativistic theory of gravity. As a result, researchers have pursued multiple avenues to investigate topics in quantum gravity theory. To date, no satisfactory quantum gravity theory has emerged to address the quantization of gravity. Fundamental contradictions between gravitational and quantum theories persist, demanding further exploration. Questions related to the quantization of gravity remain a hot topic among astrophysicists, driving meaningful and in-depth studies into diverse gravitational theories. Despite its potential in explaining quantum gravitational effects, loop quantum gravity theory still faces challenges such as the lack of experimental verification and the complexity of mathematical computations. Another fundamental approach to constructing gravitational theories assumes that Lorentz symmetry is broken at high energies but restored at low energies, consistent with current experimental results. This insight has inspired researchers to incorporate Lorentz-breaking mechanisms into studies of gravitational renormalization, such as Horava–Lifshitz gravity and Einstein-aether gravity. These theories exhibit consistency in their low-energy limits. In recent years, the effects of Lorentz-breaking on the curved spacetime background of black holes and its influence on the dynamics of bosons and fermions in black hole spacetimes have become significant research topics. However, in the realm of black hole thermodynamics, the corrections to Hawking temperature and Bekenstein–Hawking entropy due to Lorentz-breaking require further investigation. Previous studies have explored the impact of Lorentz-breaking on the Bekenstein–Hawking entropy of uncharged accelerating Kinnersley black holes and its physical implications [9]. Nevertheless, research on entropy corrections for charged accelerating AdS black holes remains insufficient. Such studies could enhance our understanding of the thermodynamic evolution characteristics of arbitrary charged, rotating, accelerating, and non-stationary black holes. According to the first law of black hole thermodynamics, Bekenstein–Hawking entropy is closely related to thermodynamic parameters such as Hawking temperature. Therefore, investigating changes in black hole entropy necessitates exploring its quantum tunneling radiation characteristics and thermodynamic evolution properties. In this paper, based on the WKB semiclassical theory, black hole quantum tunneling radiation theory, and Lorentz-breaking theory, we study the thermodynamic properties of charged accelerating AdS black holes. We also analyze the effects of Lorentz-breaking and *ℏ* perturbation theory on the quantum tunneling radiation rates of bosons and fermions, as well as the Bekenstein–Hawking entropy of these black holes.

In Section 2, we first investigate the modified form of the bosonic dynamical equations in the spacetime of a charged accelerating AdS black hole. Building upon this foundation, we analyze the corrected entropy of this charged accelerating AdS black hole and explore its physical implications. In Section 3, we extend our study to the modification of fermionic dynamical equations and examine the corresponding corrections to black hole entropy. Finally, in Section 4, we provide a detailed discussion of the methodologies employed in this work and the significance of the obtained results.

## 2. Research on Hawking Temperature and Bekenstein–Hawking Entropy of Charged Accelerating AdS Black Holes

In the study of string theory and quantum gravity theories, it has been found that the Lorentz dispersion relations require modifications at the Planck-scale level in high-energy domains [10,11,12,13,14]. Based on Einstein-aether gravity theory and Lorentz-breaking theory, the action $S_{b}$ of the scalar field φ is modified as follows:

(1) $$\begin{array}{c} S_{b}=\frac{1}{2}\int d^{4}x\sqrt{-g}\left[\left(\tilde{□}+\frac{m^{2}}{\hbar^{2}}\right)\varphi^{2}+\lambda {\left(u^{\mu }\partial_{\mu }\varphi \right)}^{2}\right] \end{array}$$

According to the computational rules of index contraction, ${\left(u^{\mu }\partial_{\mu }\right)}^{2}$ should be expressed as $u^{\mu }\partial_{\mu }u^{\nu }\partial_{\nu }$. In Equation (1), the operator $\tilde{□}$ represents the d’Alembertian operator in Riemannian space. The vector $u^{\mu }$ denotes an aether-like field vector, which satisfies the condition $u^{\mu }u_{\mu }=\alpha_{0}\left(\mathrm{const}.\right)$ in curved spacetime, where $\alpha_{0}$ is a real constant. The parameter λ is a small quantity. From a mathematical perspective, $\tilde{□}=\mathrm{div}\left(\mathrm{div}\varphi \right)$ represents the divergence of the gradient of a scalar field. According to the rules of divergence computation, we obtain

(2) $$\begin{array}{cc} \tilde{□} & =\partial_{;\mu }\left(g^{\mu \nu }\frac{\partial \varphi }{\partial x^{\nu }}\right)=\frac{\partial }{\partial x^{\mu }}\left(g^{\mu \nu }\frac{\partial \varphi }{\partial x^{\nu }}\right)+\Gamma_{\mu \nu }^{\nu }\left(g^{\mu \nu }\frac{\partial \varphi }{\partial x^{\nu }}\right)\\ \; & =\frac{\partial }{\partial x^{\mu }}\left(g^{\mu \nu }\frac{\partial \varphi }{\partial x^{\nu }}\right)+\frac{\partial }{\partial x^{\mu }}\left(\ln\sqrt{-g}\right)\left(g^{\mu \nu }\frac{\partial \varphi }{\partial x^{\nu }}\right)\\ \; & =\frac{1}{\sqrt{-g}}\frac{\partial }{\partial x^{\mu }}\left(\sqrt{-g}g^{\mu \nu }\frac{\partial \varphi }{\partial x^{\nu }}\right) \end{array}$$

In Equation (2), $\partial_{;\mu }$ denotes the covariant derivative of the contravariant vector field $\left(g^{\mu \nu }\frac{\partial \varphi }{\partial x^{\nu }}\right)=A^{\mu }$. Following the conventions of Riemannian geometry, this is computed as $A_{;\nu }^{\mu }=A_{,\nu }^{\mu }+\Gamma_{\lambda \nu }^{\mu }A^{\nu }$, where $\Gamma_{\lambda \nu }^{\mu }$ is the Christoffel symbol. Substituting this into the field expression yields Equation (2). In Equation (2), $\Gamma_{\mu \nu }^{\nu }$ represents the Christoffel symbols associated with the metric tensor of curved spacetime. Based on the variational principle and Equation (1), it can be concluded that the modified bosonic dynamical equations, accounting for Lorentz-breaking theory in curved spacetime, are determined by the following equation:

(3) $$\begin{array}{c} \delta S_{b}=0 \end{array}$$

Noting that $\delta \varphi^{2}=2\varphi \delta \varphi$, $\delta {\left(u^{\mu }\partial_{\mu }\phi \right)}^{2}=2\left(u^{\mu }\partial_{\mu }\phi \right)\left(u^{\nu }\partial_{\nu }\delta \phi \right)$ and considering that both the integral and variational operators commute, and the variational and differential operators commute, from Equations (1)–(3), the dynamical equation of the scalar field in Lorentz space, modified by Lorentz-breaking theory, is obtained as

(4) $$\begin{array}{c} \frac{1}{\sqrt{-g}}\frac{\partial }{\partial x^{\mu }}\left(\sqrt{-g}g^{\mu \nu }\frac{\partial \varphi }{\partial x^{\nu }}\right)+\lambda u^{\mu }u^{\nu }\frac{\partial }{\partial x^{\mu }}\left(\frac{\partial \varphi }{\partial x^{\nu }}\right)+\frac{m^{2}}{\hbar^{2}}\varphi =0 \end{array}$$

In fact, this is the modified form of the dynamical equation for a boson with mass *m*. If we consider the electromagnetic potential $A_{\mu }$ generated by a charged black hole with charge *q*, then the modified form of the dynamical equation for a boson with mass *m* and charge *e* is given by

(5) $$\begin{array}{c} \frac{1}{\sqrt{-g}}\left(D_{\mu }^{\prime }\sqrt{-g}g^{\mu \nu }D_{\nu }^{\prime }\right)\varphi +\lambda u^{\mu }u^{\nu }D_{\mu }^{\prime }D_{\nu }^{\prime }\varphi +\frac{m^{2}}{\hbar^{2}}\varphi =0 \end{array}$$

where $D_{\mu }^{\prime }=\frac{\partial }{\partial X^{\mu }}+\frac{ieA_{\mu }}{\hbar },D_{\nu }^{\prime }=\frac{\partial }{\partial X^{\nu }}+\frac{ieA_{\nu }}{\hbar }$. Equations (4) and (5) represent the modified forms of the dynamical equations for spin-zero bosons after incorporating Lorentz-breaking corrections. It is evident that Equation (5) includes an additional correction term compared to the Klein–Gordon equation. We can refer to this equation as the Klein–Gordon–Lorentz equation. In this equation, the correct choice of the aether-like field vector $u^{\mu }$ must be based on the characteristics of the specific black hole spacetime metric tensor $g^{\mu \nu }$. That is, $u^{\mu }$ can be appropriately selected according to the features of different curved spacetimes to ensure the validity of the research results. To illustrate the application of Equation (5) and its associated physical significance, we consider a charged accelerating AdS black hole. According to references [15,16,17,18], a charged accelerating AdS black hole is described by the metric and gauge potential

(6) $$\begin{array}{c} ds^{2}=\frac{1}{\Omega^{2}}\left[f\left(r\right)dt^{2}-\frac{dr^{2}}{f(r)}-r^{2}\left(\frac{d\theta^{2}}{g(\theta )}+g\left(\theta \right)\sin^{2}\theta \frac{d\Phi^{2}}{K^{2}}\right)\right] \end{array}$$

where

(7) $$\begin{array}{cc} f(r) & =\left(1-Ar^{2}\right)\left(1-\frac{2M}{r}+\frac{q^{2}}{r^{2}}\right)+\frac{r^{2}}{l^{2}}\\ g(\theta ) & =1+2MA\cos\theta +q^{2}A^{2}\cos^{2}\theta \end{array}$$

Conformal factor Ω is

(8) $$\begin{array}{c} \Omega =1+Ar\cos\theta . \end{array}$$

The charge *q* of this black hole and the electromagnetic potential $A_{t}$ generated by the black hole are given at the event horizon as follows:

(9) $$\begin{array}{c} \Phi =A_{t}=\frac{q}{r_{+}} \end{array}$$

To clarify the acceleration mechanism of the black hole described by Equations (6)–(9), it is essential to examine the underlying process driving this acceleration. For any physical object that interacts with the event horizon of this black hole, its energy–momentum tensor must locally satisfy $T_{0}^{0}∼T_{r}^{r}$. A physical object that meets this condition is a cosmic string. The gravitational effects of a cosmic string induce an overall global conical deficit on a spatial section perpendicular to the string. Hence, the cosmic string essentially represents a localized conical deficit in spacetime. It is precisely due to this conical deficit that the acceleration of the black hole is driven. In the above equations, Ω determines the conformal infinity or boundary of the AdS spacetime. *K* represents the conical deficit, while *M* and *q* correspond to the black hole’s mass and electric charge, respectively. A>0 is related to the magnitude of the black hole’s acceleration, and $l=\sqrt{\frac{-\Lambda }{3}}$ denotes the AdS radius. One of the notable features of this curved spacetime is that the axis passing through the black hole exhibits a deviation from local flatness. According to Equation (7), the north pole corresponds to $\theta_{+}=0$, while the south pole is at $\theta_{-}=\pi$. According to Equation (6), the regularity condition of the metric at a pole demands $K_{\pm }=g\left(\theta_{\pm }\right)=1\pm 2MA+q^{2}A^{2}$. By fixing, $K_{+}=K$ at θ=0, there is a conical deficit δ on the south pole axis at θ=π, given by $\delta =2\pi \left[1-\frac{g(\theta_{-})}{K_{+}}\right]$. The parameters in the C-metric described by Equation (6) include the black hole mass *M*, charge *q*, acceleration *A*, cosmological constant *l*, and tension of the cosmic strings on each axis, encoded by the periodicity of the angular coordinate. For this particular type of black hole, it is necessary to study its thermodynamic properties. From Equation (6), the determinant *g* of the contra variant metric and the non-zero component of the metric tensor are given as follows:

(10) $$\begin{array}{c} g=-\frac{1}{K^{2}\Omega^{2}}r^{4}\sin^{2}\theta \end{array}$$

(11) $$\begin{array}{c} g^{\mu \nu }=\left(\begin{array}{cccc} \frac{\Omega^{2}}{f(r)} & 0 & 0 & 0\\ 0 & -\Omega^{2}f\left(r\right) & 0 & 0\\ 0 & 0 & -\frac{\Omega^{2}g\left(\theta \right)}{r^{2}} & 0\\ 0 & 0 & 0 & -\frac{\Omega^{2}K^{2}}{g\left(\theta \right)r^{2}\sin^{2}\theta } \end{array}\right) \end{array}$$

The null hypersurface equation describing the black hole horizon is given by

(12) $$\begin{array}{c} g^{\mu \nu }\frac{\partial F}{\partial x^{\mu }}\frac{\partial F}{\partial x^{\nu }}=0 \end{array}$$

According to Equations (6)–(8), (11) and (12), it follows that the equation that the event horizon of the black hole in question satisfies can be obtained as $g^{rr}{\left(\frac{\partial F}{\partial r}\right)}^{2}=0$ or $\Omega^{2}f\left(r\right)=0$, i.e.,

(13) $$\begin{array}{c} \left(1+2Ar\cos\theta +A^{2}r^{2}\cos^{2}\theta \right)\left[\left(1-Ar^{2}\right)\left(1-\frac{2M}{r}+\frac{q^{2}}{r^{2}}\right)+\frac{r^{2}}{l^{2}}\right]=0. \end{array}$$

It can be seen from Equation (13) that $\Omega^{2}\neq 0$, and the equation governing the black hole event horizon $r_{+}$ is given by

(14) $$\begin{array}{c} f\left(r_{+}\right)=\left(1-Ar_{+}^{2}\right)\left(1-\frac{2M}{r_{+}}+\frac{q^{2}}{r_{+}^{2}}\right)+\frac{r_{+}^{2}}{l^{2}}=0 \end{array}$$

From Equation (7), it is evident that the acceleration parameter competes with the cosmological constant term $\frac{r^{2}}{l^{2}}$ in the Newtonian potential. Alternatively, the negative curvature of the AdS space negates the effect of acceleration. When considering the condition $A<\frac{1}{l}$ in Equations (6)–(8), we describe a single black hole suspended in AdS space, with the only horizon being that of the black hole itself [15,16,17,18,19,20,21]. When $A>\frac{1}{l}$, two oppositely charged black holes are present and separated by the acceleration horizon. The case $A=\frac{1}{l}$ corresponds to a special scenario. To ensure the validity of $K_{\pm }$, the following analysis imposes the constraint MA<1/2, which guarantees that the angular coordinates correspond to those of a standard two-sphere. With the horizon area Equation (14) and its implications established, we proceed to investigate the thermodynamic properties of this black hole. For the purpose of solving Equation (4), we select the aether-like field vector $u^{\mu }$ as follows:

(15) $$\begin{array}{c} u^{\mu }=\left(\frac{c_{t}}{\sqrt{g^{tt}}},\frac{c_{r}}{\sqrt{g^{rr}}},\frac{c_{\theta }}{\sqrt{g^{\theta \theta }}},\frac{c_{\phi }}{\sqrt{g^{\phi \phi }}}\right) \end{array}$$

Equation (15) demonstrates that $u^{\mu }u_{\mu }=c_{t}^{2}+c_{r}^{2}+c_{\theta }^{2}+c_{\phi }^{2}=c^{2}$, where $c_{t}$, $c_{r}$, $c_{\theta }$,$c_{\phi }$, and *c* are constants. Thus, the four components of $u^{\mu }$ selected here satisfy $u^{\mu }u_{\mu }=\mathrm{const}$. The WKB approximation, named after Wenzel, Kramers, and Brillouin, is a theoretical method used to solve wave function equations in quantum systems. It constructs the wave function in the form of an exponential function and expands it within the framework of semiclassical theory for solution. According to the WKB semiclassical theory, the wave function φ in Equation (15) can be expressed as

(16) $$\begin{array}{c} \varphi =\varphi_{0}e^{i\frac{S}{h}} \end{array}$$

where *S* is the action for a boson with electric charge *e* and mass *m*. Substituting Equations (15) and (16) into Equation (5) yields

(17) $$\begin{array}{c} \left(g^{\mu \nu }+\lambda u^{\mu }u^{\nu }\right)\left(\frac{\partial S}{\partial x^{\mu }}+eA_{\mu }\right)\left(\frac{\partial S}{\partial x^{\nu }}+eA_{\nu }\right)+m^{2}=0 \end{array}$$

The curved spacetime described by Equation (6) admits a Killing vector $\partial_{t}$. Therefore, we obtain

(18) $$\begin{array}{c} \frac{\partial S}{\partial t}=-\omega \end{array}$$

where ω represents the particle energy. The action *S* in Equation (17) can be separated into variables as S=−ωt+R(r)+Y(θ)+jϕ. As can be seen from Equation (9), on the event horizon of this black hole, when $r\to r_{+}$, $A_{t}=q/r_{+}$. Further simplifying Equation (17) yields

(19) $$\begin{array}{cc} \; & \frac{\Omega^{2}}{f(r)}\left(1+\lambda c_{t}^{2}\right){\left(\frac{\partial S}{\partial t}+eA_{t}\right)}^{2}-\Omega^{2}f\left(r\right)\left(1+\lambda c_{r}^{2}\right){\left(\frac{\partial S}{\partial r}\right)}^{2}+g^{\theta \theta }\left(1+\lambda c_{\theta }^{2}\right){\left(\frac{\partial S}{\partial \theta }\right)}^{2}\\ \; & +g^{\phi \phi }\left(1+\lambda c_{\phi }^{2}\right){\left(\frac{\partial S}{\partial \phi }\right)}^{2}+m^{2}=0 \end{array}$$

Since $f\left(r\right){|}_{r\to r_{+}}=0$, from this equation, we can obtain

(20) $$\begin{array}{c} {\left[f\left(r\right)\left(\frac{dR}{dr}\right)\right]}_{r\to r_{+}}^{2}=\frac{1+\lambda c_{t}^{2}}{1+\lambda c_{r}^{2}}{\left(\omega -\frac{eq}{r_{+}}\right)}^{2}=\frac{1+\lambda c_{t}^{2}}{1+\lambda c_{r}^{2}}{\left(\omega -\omega_{0}\right)}^{2} \end{array}$$

or

(21) $$\begin{array}{c} \frac{dR^{\pm }}{dr}{|}_{r\to r_{+}}=\pm {\left(\frac{1+\lambda c_{t}^{2}}{1+\lambda c_{r}^{2}}\right)}^{\frac{1}{2}}\frac{\omega -\omega_{0}}{{\left.f(r)\right|}_{r\to r_{h}}}. \end{array}$$

According to the residue theorem for integration, we obtain

(22) $$\begin{array}{c} R^{\pm }=\pm i2\pi {\left(\frac{1+\lambda c_{t}^{2}}{1+\lambda c_{r}^{2}}\right)}^{\frac{1}{2}}\frac{\omega -\omega_{0}}{f^{\prime }\left(r_{+}\right)}, \end{array}$$

where

(23) $$\begin{array}{c} f^{\prime }\left(r_{+}\right)=2\left[\frac{M}{r_{+}^{2}}-\frac{q^{2}}{r_{+}^{3}}+AM-Ar_{+}+\frac{r_{+}}{l^{2}}\right]. \end{array}$$

According to the quantum tunneling radiation theory, the quantum tunneling rate of a spin-zero, mass *m*, charge *e* boson at the event horizon of this black hole is

(24) Γ∼exp−2ImS+−ImS−=exp−2ImR+−ImR−=exp−4π1+λct21+λcr21f′(r+)(ω−ω0)=exp−ω−ω0TH,

where

(25) $$\begin{array}{c} T_{H}=\alpha \frac{f^{\prime }\left(r_{+}\right)}{4\pi }=\alpha \left(\frac{M}{2\pi r_{+}^{2}}-\frac{q^{2}}{2\pi r_{+}^{3}}+\frac{AM}{2\pi }-\frac{Ar_{+}}{2\pi }+\frac{r_{+}}{2\pi l^{2}}\right)=\alpha T_{h}. \end{array}$$

where $\alpha ={\left(\frac{1+\lambda c_{t}^{2}}{1+\lambda c_{r}^{2}}\right)}^{\frac{1}{2}}$. In Equation (20), $\omega_{0}=\frac{eq}{r_{+}}$ is the chemical potential. $T_{H}$ is the expression of the Hawking temperature at the event horizon of this black hole after being corrected by Lorentz-breaking. Obviously, the quantum tunneling rate of the bosons and the Hawking temperature at the event horizon of a uniformly accelerating charged black hole are not only related to the acceleration parameter *A* but also to the *t*-component and *r*-component of the Lorentz-breaking-related correction term $u^{\mu }$. When the aether-like correction terms are not considered, $T_{H}=T_{h}$, which is completely consistent with the result of the Hawking temperature $T_{h}$ obtained by the Euclidean method in [15,16,17]. The Bekenstein–Hawking entropy of this black hole is closely related to its Hawking temperature. If we use the condition 2mA<1 to preserve the metric signature, then *K* encodes information about the conical deficits on the north and south poles with tensions given by [22]

(26) $$\begin{array}{c} u_{\pm }=\frac{\delta_{\pm }}{8\pi }=\frac{1}{4}\left(1-\frac{1\pm 2mA+q^{2}A^{2}}{K}\right) \end{array}$$

Therefore, the first law of thermodynamics for this black hole can be expressed as

(27) $$\begin{array}{c} dM=T_{\hbar }dS_{bh}+\Phi dq+VdP-\lambda_{+}du_{+}-\lambda_{-}du_{-}, \end{array}$$

where

(28) $$\begin{array}{c} V=\frac{4}{3}\frac{\pi }{K}\left[\frac{r_{+}^{2}}{{\left(1-A^{2}r_{+}^{2}\right)}^{2}}\right], \end{array}$$

In Equation (27), $\lambda_{\pm }$ denotes the thermodynamic lengths associated with the tension charges of the strings, corresponding to the segments of string attached at each pole. The expression for $\lambda_{\pm }$ is given as follows:

(29) $$\begin{array}{c} \lambda_{\pm }=\frac{r_{+}}{1\pm Ar_{+}}-M\left(1\pm 2MA+q^{2}A^{2}\right). \end{array}$$

Here, *V* represents the black hole thermodynamic volume, and *P* is the pressure associated with the cosmological constant. From Equation (27), we can obtain

(30) $$\begin{array}{cc} S_{BH} & =\int \frac{dM-Vdp-\phi dq+\lambda_{+}du_{+}+\lambda_{-}du_{-}}{T_{H}}\\ \; & ={\left(\frac{1+\lambda c_{t}^{2}}{1+\lambda c_{r}^{2}}\right)}^{-\frac{1}{2}}\int \frac{dM-Vdp-\phi dq+\lambda_{+}du_{+}+\lambda_{-}du_{-}}{T_{h}}\\ \; & ={\left(\frac{1+\lambda c_{t}^{2}}{1+\lambda c_{r}^{2}}\right)}^{-\frac{1}{2}}S_{bh}, \end{array}$$

In the research on black hole entropy, quantum gravitational effects induce Lorentz symmetry breaking without altering the Bekenstein–Hawking entropy of the black hole. When considering *ℏ*-perturbation theory, logarithmic corrections to the black hole entropy arise. In the present study, we have modified the quantum tunneling rate of this black hole via Lorentz-breaking theory, thereby obtaining the corrected Hawking temperature at the event horizon of the black hole. Consequently, based on the first law of black hole thermodynamics, the modified Bekenstein–Hawking entropy expression as shown in Equation (30) is derived. $S_{BH}$ is the result of the Lorentz-breaking correction to the Bekenstein–Hawking entropy of this black hole, and $S_{bh}$ is the Bekenstein–Hawking entropy of this black hole without considering Lorentz-breaking. Here,

(31) $$\begin{array}{c} S_{bh}=\frac{a_{s}}{4}. \end{array}$$

where $a_{s}$ is the area of the event horizon of this black hole. As can be seen from Equation (6), $dS^{\prime }=\frac{1}{\Omega^{2}}\left(\frac{r^{2}}{g(\theta )}d\theta^{2}-\frac{g\left(\theta \right)r^{2}\sin^{2}\theta }{K^{2}}d\phi^{2}\right)$. Therefore, we have

(32) $$\begin{array}{c} a_{s}=\int d\theta d\phi \sqrt{-g^{\prime }}=\frac{2\pi r_{+}^{2}}{K}\int_{0}^{\pi }\frac{\sin^{2}\theta d\theta }{{\left(1+Ar_{+}\cos\theta \right)}^{2}}=\frac{4\pi r_{+}^{2}}{K}\frac{1}{1-A^{2}r_{+}^{2}}. \end{array}$$

Substituting Equation (32) into Equation (31), we obtain that the expression of the Bekenstein–Hawking entropy of this black hole before correction is

(33) $$\begin{array}{c} S_{bh}=\frac{\pi r_{+}^{2}}{K\left(1-A^{2}r_{+}^{2}\right)}. \end{array}$$

Therefore, Equation (30) can be rewritten as

(34) $$\begin{array}{c} S_{BH}={\left(\frac{1+\lambda c_{t}^{2}}{1+\lambda c_{r}^{2}}\right)}^{-\frac{1}{2}}\frac{\pi r_{+}^{2}}{K\left(1-A^{2}r_{+}^{2}\right)}. \end{array}$$

$T_{H}$ and $S_{BH}$ are the corrected results of the Hawking temperature and the Bekenstein–Hawking entropy of this black hole obtained within the semi-classical theory. To demonstrate the effect of the quantum perturbation theory on the correction of the Bekenstein–Hawking entropy of this black hole, we first rewrite Equation (21) and its solution Equation (22) as

(35) $$\begin{array}{cc} {\left.\frac{dR_{0}^{\pm }}{dr}\right|}_{r\to r_{+}} & =\pm {\left(\frac{1+\lambda c_{t}^{2}}{1+\lambda c_{r}^{2}}\right)}^{\frac{1}{2}}\frac{E_{0}}{{\left.f(r)\right|}_{r\to r_{+}}} \end{array}$$

(36) $$\begin{array}{cc} R_{0}^{\pm } & =\pm i\pi {\left(\frac{1+\lambda c_{t}^{2}}{1+\lambda c_{r}^{2}}\right)}^{\frac{1}{2}}\frac{E_{0}}{f^{\prime }\left(r_{+}\right)}, \end{array}$$

where $E_{0}=\omega -\omega_{0}$. Considering the quantum perturbation theory, we can express the energy and the radial action of bosons in the spacetime of this black hole as follows:

(37) $$\begin{array}{c} \tilde{E}=E_{0}+\sum_{i=1}\hbar^{i}E_{i} \end{array}$$

(38) $$\begin{array}{c} {\tilde{R}}^{\pm }=R_{0}^{\pm }+\sum_{i=1}\hbar^{i}R_{i}^{\pm } \end{array}$$

Substituting i=1,2,3,… into Equation (20), respectively, we obtain

(39) $$\begin{array}{c} {\left.\frac{dR_{1}^{\pm }}{dr}\right|}_{r\to r_{+}}=\pm {\left(\frac{1+\lambda c_{t}^{2}}{1+\lambda c_{r}^{2}}\right)}^{\frac{1}{2}}\frac{E_{1}}{{\left.f(r)\right|}_{r\to r_{+}}} \end{array}$$

(40) $$\begin{array}{c} {\left.\frac{dR_{2}^{\pm }}{dr}\right|}_{r\to r_{+}}=\pm {\left(\frac{1+\lambda c_{t}^{2}}{1+\lambda c_{r}^{2}}\right)}^{\frac{1}{2}}\frac{E_{2}}{{\left.f(r)\right|}_{r\to r_{+}}}. \end{array}$$

Evidently, there exists an inevitable connection between $R_{1}^{\pm },R_{2}^{\pm },\ldots$ and $R_{0}^{\pm }$. That is to say, if we denote $\frac{R_{i}^{\pm }}{R_{i-1}^{\pm }}$ as $\alpha_{i}^{\prime }=\frac{\alpha_{i}}{S_{BH}}$, then after the quantum perturbation correction, the quantum tunneling rate of bosons at the event horizon of this black hole is further modified to

(41) Γ˜∼exp−2ImR˜i+−ImR˜i−=exp−4π1+λct21+λcr212E0f′r+1+∑i=1ℏiαi′=exp−E0TH˜,

where

(42) $$\begin{array}{c} \tilde{T_{H}}=\frac{f^{\prime }\left(r_{+}\right)}{4\pi }{\left(\frac{1+\lambda c_{r}^{2}}{1+\lambda c_{t}^{2}}\right)}^{\frac{1}{2}}\left(1-\sum_{i=1}\hbar^{i}\alpha_{i}^{\prime }\right). \end{array}$$

Therefore, when quantum perturbation corrections are considered, the Bekenstein–Hawking entropy of this black hole can be further expressed as

(43) $$\begin{array}{c} \tilde{S_{BH}}=S_{BH}+\hbar \beta_{1}\ln S_{BH}+\ldots \end{array}$$

where $\beta_{1}$ is the proportionality coefficient between option $\hbar^{1}$ and $\hbar^{0}$. This perturbatively corrected result indicates that $\tilde{S_{BH}}$ has a logarithmic correction [23,24]. The derived black hole entropy demonstrates that it depends not only on the event horizon area but also on the quantum parameter $\hbar^{i}$ and the coefficients $c_{t}$, $c_{r}$ of the Lorentz-breaking correction terms. These findings highlight the complexity of black hole entropy, which remains a topic warranting further investigation.

## 3. Lorentz-Breaking Corrections to Quantum Tunneling Radiation of Spin-1/2 Fermions in Accelerating Charged AdS Black Hole Spacetime

In flat spacetime, the action of a spinor field can be expressed in the following two forms [25]:

(44) $$\begin{array}{c} \bar{S}=\int d^{4}x\bar{\psi^{\prime }}\left[i\gamma^{m}\left(\partial_{m}-ieA_{m}\right)-m\right]\psi^{\prime } \end{array}$$

(45) $$\begin{array}{c} {\bar{S}}^{\prime }=\int d^{4}x\bar{\psi^{\prime }}\left[i\gamma^{m}\left(\partial_{m}-ieA_{m}\right)\left(1-\alpha \frac{\gamma^{m}D_{m}}{m^{2}}+b\gamma^{5}+\xi {\left(bD\right)}^{2}\right)-m\right]\psi^{\prime }. \end{array}$$

In Equation (45), $D_{m}=\partial_{m}-ieA_{m}$ denotes the standard gauge covariant derivative. Equation (44) represents the action without incorporating Lorentz-breaking corrections, while ${\bar{S}}^{\prime }$ corresponds to the action including such corrections. Here, $\bar{\psi^{\prime }}$ is the complex conjugate of $\psi^{\prime }$, applicable for spin-1/2 fermions. Since spin-1/2 fermions can have both spin-up and spin-down states, a chiral correction term, $b\gamma^{5}$, must be introduced into Equation (45) when considering Lorentz-breaking effects. Additionally, the first known Lorentz-breaking term is the Carroll–Field–Jackiw (CFJ) term, which was proposed and explicitly calculated in [26,27]. Therefore, the action of the spinor field must also include the CFJ correction term. In summary, in the curved spacetime of a black hole, considering the Lorentz-breaking theory, we can modify the spinor field action. We propose that the action of the spinor field in curved spacetime be modified to the following form [25]:

(46) $$\begin{array}{cc} S_{f} & =\int d^{4}x\sqrt{-g}\bar{\psi }\left\{i\gamma^{\mu }D_{\mu }\left[1-\frac{a\hbar^{2}}{m^{2}}\gamma^{\mu }\gamma^{\nu }D_{\mu }D_{\nu }\right]+\left(\frac{\lambda }{m}\right)\hbar u^{\mu }u^{\nu }D_{\mu }D_{\nu }+\left(\frac{b}{m}\right)\frac{\gamma^{5}}{\hbar }-\frac{m}{\hbar }\right\}\psi \\ \; & =\int d^{4}x\sqrt{-g}L_{f} \end{array}$$

where $D_{\mu }=\partial_{\mu }+\frac{i}{\hbar }eA_{\mu }+\Omega_{\mu }$, $\Omega_{\mu }=\frac{i}{2}\Gamma_{\mu }^{\alpha \beta }\Pi_{\alpha \beta }$ and $\Pi_{\alpha \beta }=\frac{i}{4}\left[\gamma^{\alpha },\gamma^{\beta }\right]$. In Equation (46), $\gamma^{\mu }$ denotes the gamma matrices in curved spacetime, which are determined by the specific characteristics of the curved spacetime, and the wave function $\bar{\psi }$ is the conjugate of ψ. The coefficient *a* represents the CFJ correction term and satisfies the condition a/m≪1. The coefficient *b* represents the chiral correction term and satisfies the condition b/m≪1. The coefficient λ represents the aether-like correction term and satisfies the condition λ/m≪1. The gamma matrices in Equation (46) satisfy the following conditions:

(47) $$\begin{array}{c} \gamma^{\mu }\gamma^{v}+\gamma^{v}\gamma^{\mu }=2g^{\mu v}I \end{array}$$

(48) $$\begin{array}{c} \gamma^{\mu }\gamma^{5}+\gamma^{5}\gamma^{\mu }=0 \end{array}$$

In the semiclassical WKB theory, the wave function ψ of a spin-1/2 fermion can be expressed as

(49) ψ=CDeiSfh.

where $S_{f}$ denotes the action of a fermion with charge *e*, mass *m*, and spin-1/2. The coefficient of ψ is a 2×1 matrix. According to the variational principle, we can obtain

(50) $$\begin{array}{c} \delta S_{f}=\int d^{4}x\sqrt{-g}\delta L_{f}=0. \end{array}$$

From Equations (45)–(48) and (51), the modified form of the dynamical equation for a spin-1/2 fermion is obtained as follows:

(51) $$\begin{array}{c} \left[g^{\mu \nu }\left(1-2a\right)+2\lambda u^{\mu }u^{\nu }\right]\left(\partial_{\mu }S_{f}+eA_{\mu }\right)\left(\partial_{\nu }S_{f}+eA_{\nu }\right)+2b\gamma_{0}^{5}+m^{2}=0 \end{array}$$

where $\gamma_{0}^{5}$ is the coefficient corresponding to $\gamma^{5}$, which must be determined based on the specific characteristics of the curved spacetime. From Equation (6), we can identify the matrix elements of the $\gamma^{\mu }$ matrices corresponding to the black hole spacetime as follows:

(52) $$\gamma^{t}=\sqrt{g^{tt}}\left(\begin{array}{cc} I & 0\\ 0 & -I \end{array}\right)=\frac{\Omega }{\sqrt{f(r)}}\left(\begin{array}{cc} I & 0\\ 0 & -I \end{array}\right)\;\gamma^{r}=\sqrt{g^{rr}}\left(\begin{array}{cc} 0 & \alpha^{1}\\ \alpha^{1} & 0 \end{array}\right)=\Omega \sqrt{-f(r)}\left(\begin{array}{cc} 0 & \alpha^{1}\\ \alpha^{1} & 0 \end{array}\right)\;\gamma^{\theta }=\sqrt{g^{\theta \theta }}\left(\begin{array}{cc} 0 & \alpha^{2}\\ \alpha^{2} & 0 \end{array}\right)=\frac{\Omega }{r}\sqrt{-g(\theta )}\left(\begin{array}{cc} 0 & \alpha^{2}\\ \alpha^{2} & 0 \end{array}\right)\;\gamma^{\phi }=\sqrt{g^{\phi \phi }}\left(\begin{array}{cc} 0 & \alpha^{3}\\ \alpha^{3} & 0 \end{array}\right)=\frac{1}{r\sin\theta }\frac{1}{\sqrt{-g(\theta )}}K\Omega \left(\begin{array}{cc} 0 & \alpha^{3}\\ \alpha^{3} & 0 \end{array}\right)$$

The Pauli matrices in Equation (51) are given by

(53) $$\alpha^{1}=\left(\begin{array}{cc} 0 & 1\\ 1 & 0 \end{array}\right)\;\alpha^{2}=\left(\begin{array}{cc} 0 & -i\\ i & 0 \end{array}\right)\;\alpha^{3}=\left(\begin{array}{cc} 1 & 0\\ 0 & -1 \end{array}\right)$$

Based on Equations (6) and (51), the choice of $\gamma^{5}$ is given by

(54) $$\begin{array}{cc} \gamma^{5} & =-K\Omega \frac{\sin\theta }{r}\gamma^{t}\gamma^{r}\gamma^{\theta }\gamma^{\phi }\\ & =-K\Omega \frac{1}{r}\left(\begin{array}{cc} I & 0\\ 0 & -I \end{array}\right)\left(\begin{array}{cc} 0 & \alpha^{1}\\ \alpha^{1} & 0 \end{array}\right)\left(\begin{array}{cc} 0 & \alpha^{2}\\ \alpha^{2} & 0 \end{array}\right)\left(\begin{array}{cc} 0 & \alpha^{3}\\ \alpha^{3} & 0 \end{array}\right)\\ & =\gamma_{0}^{5}\left(\begin{array}{cc} 1 & 0\\ 0 & 1 \end{array}\right) \end{array}$$

where $\gamma_{0}^{5}=K\Omega /r$, and it is clear that $\gamma^{5}$ satisfies Equation (48). The $\gamma^{\mu }$ and $\gamma^{5}$ chosen from Equations (52)–(54) fully satisfy Equations (47) and (48). Moreover, $\gamma^{t}$, $\gamma^{r}$, $\gamma^{\theta }$, $\gamma^{\phi }$, and $\gamma^{5}$ are all Hermitian matrices, ensuring that the related calculations have physical significance. By substituting Equations (11), (15) and (54) into the fermion dynamical Equation (51), Equation (51) is specifically given by

(55) $$\begin{array}{c} \begin{array}{cc} \; & \frac{\Omega^{2}}{f(r)}\left(1-2a+2\lambda c_{t}^{2}\right){\left(\frac{\partial S_{f}}{\partial t}+eA_{t}\right)}^{2}-\Omega^{2}f\left(r\right)\left(1-2a+2\lambda c_{r}^{2}\right){\left(\frac{\partial S_{f}}{\partial r}\right)}^{2}-\frac{\Omega^{2}g\left(\theta \right)}{r^{2}}(1-2a+\\ \; & \left.2\lambda c_{\theta }^{2}\right){\left(\frac{\partial S_{f}}{\partial \theta }\right)}^{2}-\frac{\Omega^{2}K^{2}}{g\left(\theta \right)r^{2}\sin^{2}\theta }\left(1-2a+2\lambda c_{\phi }^{2}\right){\left(\frac{\partial S_{f}}{\partial \phi }\right)}^{2}+\frac{2bK\Omega }{r}+m^{2}=0 \end{array} \end{array}$$

The curved spacetime described by the metric Equation (6) possesses Killing vectors $\partial_{t}$ and $\partial_{\varphi }$. Therefore, the action in Equation (55) can be separated as

(56) $$\begin{array}{c} S_{f}=-\omega t+R_{f}\left(r\right)+Y\left(\theta \right)+j\phi \end{array}$$

Substituting Equation (56) into Equation (55) and multiplying both sides of the equation by ${\left.f(r)\right|}_{r\to r_{H}}$, we obtain

(57) $$\begin{array}{c} {\left.\frac{dR_{f}^{\pm }}{dr}\right|}_{r\to r_{H}}=\pm \frac{1}{{\left.f(r)\right|}_{r\to r_{H}}}{\left(\frac{1-2a+2\lambda c_{t}^{2}}{1-2a+2\lambda c_{r}^{2}}\right)}^{1/2}\left(\omega -\omega_{0}\right). \end{array}$$

Using the residue theorem and setting $R_{f}^{\pm }=\tilde{R_{0}^{\pm }}$, we obtain

(58) $$\begin{array}{c} \tilde{R_{0}^{\pm }}=\pm \frac{i2\pi }{f^{\prime }\left(r_{t}\right)}{\left(\frac{1-2a+2\lambda c_{t}^{2}}{1-2a+2\lambda c_{r}^{2}}\right)}^{1/2}\left(\omega -\omega_{0}\right). \end{array}$$

This yields the quantum tunneling rate of spin-1/2 fermions at the event horizon of a charged, accelerating AdS black hole:

(59) Γf∼exp−2ImR0+˜−ImR0−˜=exp−4π1−2a+2λct21−2a+2λcr21/2ω−ω0f′r+=exp−ω−ω0TH′˜

where $f^{\prime }\left(r_{+}\right)$ is given by Equation (23). The corresponding expression for $\tilde{T_{H}^{\prime }}$ is provided below:

(60) $$\begin{array}{cc} \tilde{T_{H}^{\prime }} & ={\left(\frac{1-2a+2\lambda c_{r}^{2}}{1-2a+2\lambda c_{t}^{2}}\right)}^{1/2}\left(\frac{M}{2\pi r_{+}^{2}}-\frac{q^{2}}{2\pi r_{+}^{3}}+\frac{AM}{2\pi }-\frac{Ar_{+}}{2\pi }+\frac{r_{+}}{2\pi l^{2}}\right)\\ \; & =T_{h}{\left(\frac{1-2a+2\lambda c_{r}^{2}}{1-2a+2\lambda c_{t}^{2}}\right)}^{1/2} \end{array}$$

This is the Hawking temperature at the event horizon of the black hole, corrected by Lorentz symmetry breaking. The correction is derived from the modified dynamical equations for spin-1/2 fermions, and it naturally differs from the corresponding result for bosons. The difference primarily manifests in the coefficients of the correction terms. According to the first law of black hole thermodynamics, the black hole entropy is related to the Hawking temperature and other factors. Based on the first law of black hole thermodynamics and *ℏ* perturbation theory, we can derive the expression for the black hole entropy, which involves terms related to $\hbar^{i}$, *a*, $c_{t}$, $c_{r}$:

(61) $$\begin{array}{c} \tilde{S_{BH}^{\prime }}=S_{BH}^{\prime }+\hbar \ln S_{BH}^{\prime }+\ldots \end{array}$$

where

(62) $$\begin{array}{c} S_{BH}^{\prime }={\left(\frac{1-2a+2\lambda c_{t}^{2}}{1-2a+2\lambda c_{r}^{2}}\right)}^{-1/2}S_{bh} \end{array}$$

Comparing with Equation (45), it is evident that $\tilde{S_{BH}^{\prime }}$ contains an additional coefficient for the CFJ correction term compared to $\tilde{S_{BH}}$. This correction arises from the spin characteristics of spin-1/2 fermions. It is important to note that the coefficient *b* of the chiral correction does not affect $\tilde{S_{BH}^{\prime }}$ as the equation for $R^{\prime }\left(r\right)$ does not exhibit any *b*-dependent correction term as $r\to r_{+}$ due to the condition $f(r_{+})b\Omega /r=0$. However, for $r>r_{+}$, the fermion energy level distribution in the black hole spacetime will inevitably include corrections that depend on *b*.

## 4. Discussion

In the above studies, we considered the case where A>0, which is related to the magnitude of the black hole’s acceleration. We applied Lorentz-breaking theories to modify the dynamical equations of bosons and fermions in the spacetime of the black hole. Based on these modifications, we introduced both Lorentz-breaking and quantum perturbation corrections to the Bekenstein–Hawking entropy of the black hole. The results obtained in this work provide valuable insights for studying other types of charged accelerating black holes. As discussed, by setting M=0 and q=0, the black hole horizon is removed, resulting in pure AdS spacetime. This allows for the study of the Rindler horizon for a uniformly accelerating observer. Similarly, the Lorentz-breaking corrections to the Hawking temperature at the Rindler horizon can be explored, along with the correction to the Bekenstein–Hawking entropy. Additionally, we can introduce Rindler-type coordinate transformations in pure AdS spacetime to further investigate the corrections to the Hawking temperature and other physical quantities at the Rindler horizon.The spacetime metric of the black hole is a solution to the Einstein–Maxwell field equation. This solution is a direct generalization of the Reissner–Nordström solution in general relativity and the Bora solution in classical electrodynamics. The results indicate that, except for an additional “outer” Killing horizon due to the accelerated motion, the horizon structure closely resembles that of the Reissner–Nordström case. Kinnersley et al. studied the spacetime characteristics of uniformly accelerating charged black holes and the spacetime metrics of black holes with arbitrary acceleration and no charge [28,29]. Black holes are possibly the most fascinating objects in the study of cosmology and astrophysics. 

## Data Availability

Data is contained within the article.
